# Improving the Design of a MscL-Based Triggered Nanovalve

**DOI:** 10.3390/bios3010171

**Published:** 2013-03-19

**Authors:** Irene Iscla, Christina Eaton, Juandell Parker, Robin Wray, Zoltán Kovács, Paul Blount

**Affiliations:** 1 Department of Physiology, University of Texas Medical Center at Dallas, 5323 Harry Hines Boulevard, Dallas, TX 75390, USA; E-Mail: irene.iscla@utsouthwestern.edu (I.I.); christina.eaton@utsouthwestern.edu (C.E.); juandell.parker@utsouthwestern.edu (J.P.); robin.wray@utsouthwestern.edu (R.W.); 2 Advanced Imaging Research Center, University of Texas Medical Center at Dallas, 5323 Harry Hines Boulevard, Dallas, TX 75390, USA; E-Mail: zoltan.kovacs@utsouthwestern.edu

**Keywords:** drug-delivery, nanovalve, osmoregulation, biosensor, hydrophobic gating, mechanosensor

## Abstract

The mechanosensitive channel of large conductance, MscL, has been proposed as a triggered nanovalve to be used in drug release and other nanodevices. It is a small homopentameric bacterial protein that has the largest gated pore known: greater than 30 Å. Large molecules, even small proteins can be released through MscL. Although MscL normally gates in response to membrane tension, early studies found that hydrophilic or charged residue substitutions near the constriction of the channel leads to pore opening. Researchers have successfully changed the modality of MscL to open to stimuli such as light by chemically modifying a single residue, G22, within the MscL pore. Here, by utilizing *in vivo*, liposome efflux, and patch clamp assays we compared modification of G22 with that of another neighboring residue, G26, and demonstrate that modifying G26 may be a better choice for triggered nanovalves used for triggered vesicular release of compounds.

## 1. Introduction

**Figure 1:**
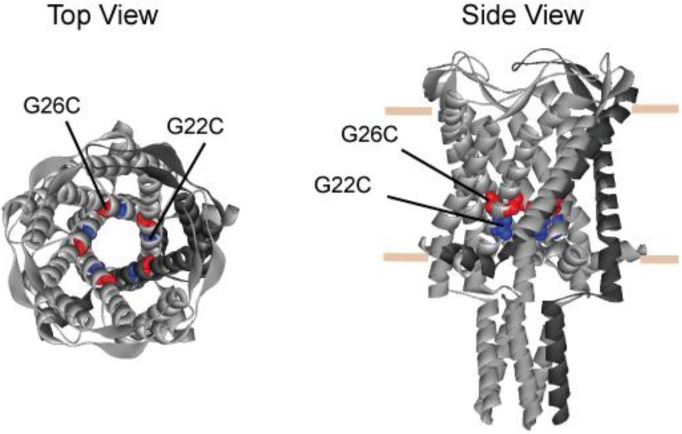
Schematic representation of MscL derived from the crystal structure of *M. tuberculosis*. The figure shows the structure of TB MscL from top (**left**) and side (**right**) views. The position of residues A20 and G24 (G22C and G26C in *E. coli* MscL, respectively) are shown as CPK in the pentameric structure. The predicted position of the lipid leaflet is indicated by horizontal bars in the side view. Note that G26 is higher in the vestibule, while G22 is more buried.

The bacterial mechanosensitive channel of large conductance, MscL, has been proposed as a triggered nanovalve for drug release as well as other nanodevices. Among its advantages is the extended structural and functional information available. Indeed, MscL of *E. coli* is one of the best studied mechanosensitive channels [1,2]. The physiological role of MscL in bacteria is osmoregulation, acting as an emergency release valve in response to a hypo-osmotic shock [3]. When a sudden decrease in the osmolarity of the environment occurs, water enters the bacterial cell producing cell swelling and creating tension in the membrane; MscL then opens a large pore, estimated to be greater than 30 Å [4], in response to membrane tension and allows the rapid flow of solutes to prevent cell lysis. The crystal structure of MscL from the homolog *M. tuberculosis* has been solved at a resolution of 3.5 Å [5,6] (Figure 1). Each subunit consists of two transmembrane (TM) domains with both the C and N terminals cytoplasmic, the TM1 domain lines the pore while TM2 is in direct contact with the lipid environment. The homopentameric structure is thought to represent a closed state, although it may not reflect the totally closed state *in vivo* [2,7,8].

Early in the study of *E. coli* MscL, because of its large pore size it was predicted that bacteria expressing MscL channels that opened inappropriately, at lower tensions, would have reduced growth or viability; this is genetically defined as a gain of function (GOF) phenotype. Random mutagenesis experiments using this GOF phenotype as the output for the screens led to the discovery of a cluster of mutations in TM1, in what is now known to be the pore constriction point [9]. A more detailed study in which the glycine at position 22 (G22) was mutated to all 19 other amino acids found that while hydrophobic substitutions led to channels that required more energy to gate, hydrophilic substitutions led to channels with lower pressure thresholds; some even gated spontaneously [10]. We later speculated that this was due to the disruption of a hydrophobic lock within the pore that stabilizes the closed state [11]. Subsequently, several studies using *E. coli* MscL cysteine mutants have shown that post-translationally adding a charge in the pore region of the channel by treatments with charged methanethiosulfonate (MTS) reagents can gate the channels in the absence of pressure [12,13,14,15]. 

These data combined with MscL’s large pore size [4] as well as the ability to isolate large amounts of protein from cells [16], cell free systems [17], or even synthesize the entire protein and yet functionally reconstitute it [18], seemed to make it an ideal candidate as a triggered nanovalve that could be used for targeted drug release or other nanotech devices. Indeed, Koçer and colleagues used these observations to modify the modality of MscL, changing it into a pH or light triggered nanovalve. This was achieved by a substitution at the well-studied G22 site to cysteine, and subsequent treatment with sulfhydryl reagents that added pH or light sensitive compounds that became charged upon decreasing pH changes or light pulses [19,20,21]. Subsequently, we have shown that MscL pore size and flux selectivity can also be controlled, further adding to the malleability of MscL as a nanovalve [22,23]. However, the choice of G22 as the site of modification is largely historical. Although G22 was arbitrarily selected for further detailed evaluation [10,15], several residues now known to be within or adjacent to the pore in the *E. coli* MscL, ranging from R13 to G30, were identified in the original forward genetics study described above [9]. Hence, other sites may be viable options.

One of the most likely alternatives to modifying the G22 site, based on existing data and models for MscL gating, would be G26 because a more recent study suggested it achieves a more open conformation upon the addition of charges at this site [13]. Here we present a series of *in vivo*, patch clamp, and efflux experiments that compare the relative efficiency of gating *E. coli* MscL through modifications at position G22 *vs*. G26. G26 MscL showed more accessibility to stimuli and is more efficient in efflux experiments, thus our results suggest that in many instances modification of MscL at this position would lead to a more efficiently triggered nanovalve for its use in targeted drug release systems. 

## 2. Experimental Section

### 2.1. *In Vivo* Viability Experiments

*E. coli* strain MJF 455 [3] was use as a host for wild type (WT), G22C and G26C MscL constructs in the pB10d expression plasmid [24]. Cells were grown in citrate-phosphate defined media with 1mM ampicillin. Overnight cultures were diluted 1:400 and grown at 37 °C in an incubator shaking at 225 rpm. When cultures reached an optical density at 600 nm (OD_600_) of approximately 0.2 they were induced for 30 min with isopropyl β-D-1-thiogalactopyranoside (IPTG). After taking OD_600_ measurements for viability calculations, samples were incubated for 15 min in the same isotonic media with or without 1 mM MTSET^+^ or 1 mM MTSES^-^. Serial dilutions of each sample were performed and 5 µL drops of each dilution were plated to determine cell viability. All MTS reagents were purchased from Toronto Research Chemicals (Toronto, ON, Canada). 

### 2.2. Electrophysiology

*E. coli* giant spheroplasts were generated as previously described [16]. Patch clamp experiments were performed in the inside-out configuration, at room temperature under symmetrical conditions using a buffer consisting of 200 mM KCl, 90 mM MgCl_2_, 10 mM CaCl_2_, and 5 mM HEPES adjusted to pH 6.0. Patches were excised, and recordings were performed at 20 mV (for simplicity the patch traces openings are shown upward). Data were acquired at a sampling rate of 20 kHz with 10 kHz filtration using an AxoPatch 200B amplifier (Molecular Devices, Sunnyvale, CA, USA). A piezoelectric pressure transducer (World Precision Instruments, Sarasota, FL, USA) was used to measure the pressure throughout the experiments.

### 2.3. Calcein Efflux Assay

The calcein efflux assay was performed as previously [25]. Constructs of WT, G22C and G26C in pet21a plasmid were used and hosted in PB116 *E. coli* strain [26]. We used a C-terminal truncated version of the G26C MscL protein (Δ110-136) because it shows about a 2 fold increase in expression when compared to the full length version (protein concentration truncated/full length 1.95 ± 0.16, n = 4). Previous experiments have demonstrated that this c-terminal truncation has no effect on channel function [26]. Overnight bacterial cultures were started by inoculating 10 mL LB media supplemented with 1mM ampicillin with a single colony from freshly streaked plates. Bacteria were grown at 37 °C in an incubator shaking at 225 rpm. Overnight cultures were diluted 1:150 in 2 liter flasks containing 1 liter of the same media. When cultures reached an OD_600_ of 0.6 for WT and G22C MscL or and an OD_600_ of 0.8 for G26C full length and G26C (Δ110-136), they were induced with IPTG for 3 hours. Cells were harvested via centrifugation at 4,000 ×*g* for 30 min. MscL proteins were purified as previously described [27]. A comparison of the protein yield for wild type (WT), G22C, G26C and G26C (Δ110-136) MscL is shown below in Table 1. The experiments were done side by side, adjusted for pellet weight, and the protein concentration was measured using a Micro BCA Protein Assay (Pierce, Rockford, Illinois) following manufacturer’s instructions. Note that almost 1 mg of the G26C (Δ110-136) MscL protein is obtained per liter of culture. Protein was reconstituted into lipid vesicles composed of 5 mg DOPC and 5 mg cardiolipin (Avanti Polar Lipids, Inc., Alabaster, AL, USA) at a protein:lipid ratio of 1:120. Briefly, lipid vesicles were passed through a lipid extruder (0.4 µM) for size homogenization and Anapoe-X-100 (Anatrace Inc.) was added to destabilize the liposomes. Then 83 µg of MscL protein was added, and vesicles were warmed to 60 °C for 30 min. Calcein was then added to a final concentration of 100 mM, mixed, and allowed to equilibrate with the liposomes. The detergent was removed by biobeads that were incubated, while mixing, overnight at 4 °C. Free calcein was then removed by passage through a G-50 fine Sephadex column (GE Healthcare Inc., Piscataway, NJ, USA), washed with vesicle buffer (10 mM NaPi, pH 8.0; 300 mM NaCl; and 1 mM EDTA), and 0.5 M sucrose was added into vesicle fractions for storage.

**Table 1:** Protein yield comparison of MscL purification.

MscL	Protein concentration, µg/mL	SEM	n
WT	2,308.3	431.8	4
G22C	1,990.1	324.1	4
G26C	454.7	104.7	4
G26C (Δ110-136)	918.6	231.8	4

Vesicles (20 µL) were placed into 180 µL of vesicle buffer with 0.5 M sucrose in clear 96-well plates. Fluorescence was recorded at 538 nm with the excitation at 485 nm using a Fluoroskan Ascent (Thermo Scientific Inc., Waltham, MA, USA). The baseline of samples was recorded for 5 min and, for treated samples, followed by the addition of 1 mM MTSET^+^. Fluorescence was monitored for at least 30 min and then vesicles were finally lysed by the addition of 10 µL of 10% Triton X-100 to determine the total released fluorescence levels. 

For flux experiments with the light sensitive compound, MscL was modified with 3*'*,3*'*-dimethyl-1*'*-(2-iodoacetyloxyethyl)-6-nitrospirol[2H-1-benzopyran-2,2'-indoline] [20], while bound to the Ni-NTA matrix by incubation at RT for 45 min at a concentration of 2 mM. The modified protein was then incorporated to the lipid vesicles and the vesicles loaded with calcein as described above. MscL gating was elicited by 5 min pulses of UV irradiation at 366 nm. Vesicles were finally lysed by the addition of 10 µL of 10% Triton X-100 to determine the total released fluorescence levels. 

## 3. Results and Discussion

### 3.1. Comparing Site Accessibility of the MscL Nanovalve *in Vivo*

**Figure 2:**
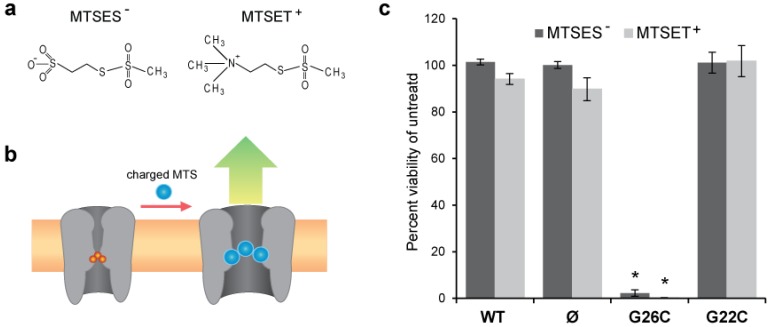
Effects of posttranslational modifications with methanethiosulfonate (MTS) reagents on G26C and G22C MscL function *in vivo.* (**a**) The chemical structures of the MTS reagents MTSES^−^ and MTSET^+^ are shown. (**b**) A scheme depicting the opening of the MscL nanovalve when a charged MTS reagents access the cysteine in the constriction pore. (**c**) A graph showing the change in viability of bacteria after incubation with charged MTS reagents. The values shown in the bar graphs represent the changes in survival rate of bacteria carrying an empty vector or expressing WT, G26C or G22C MscL when incubated for 15 min with 1 mM MTSET^+^ or 1mM MTSES^−^ reagents. Values are the average of at least three experiments and the error bars represent the standard error of the mean (SEM), n = 6, * p ≤ 2 × 10^−7 ^Student-t-test unpaired WT *vs*. G26C MscL.

One of the major factors in determining the relative efficacy of the two sites for triggering the MscL-based nanovalve is the accessibility of the sites to modification as well as the stimuli that would be required for MscL pore opening. Because G26 is higher in the vestibule of the pore, while G22 is largely buried, as shown in Figure 1, we hypothesized that G26 would be more malleable. To quickly compare the ability to manipulate residues at position G22 and G26, we designed a series of *in vivo* experiments. We used cell viability as an output to evaluate the relative efficiency of modifications with charged methanethiosulfonate (MTS) reagents to induce phenotypic changes in MscL cysteine mutants G22C and G26C. Briefly, the viability of *E. coli* bacteria was measured after incubation in the presence or absence of the charged MTS reagents MTSES^-^ or MTSET^+^, whose structures are shown in Figure 2(a). If a charged MTS reagent accesses and binds to the cysteine in the constriction pore of MscL (Figure 2(b)), the channel gates compromising the viability of the bacteria. The results of these experiments, expressed as percentage viability of the control (untreated), are shown in the graph in Figure 2(c), where the changes in viability of bacteria carrying an empty plasmid, or expressing WT, G26C, or G22C MscL are compared. 

Bacteria expressing G26C MscL were the only group to show a severe decrease in viability after treatment with both MTS reagents. Since the cells were not osmotically shocked while incubated with the MTS reagents, we assume that the channels were in a closed state. Yet, a previous study has shown that when the G22C MscL mutant is gated by osmotic shock, this mutant is sensitive to MTSET^+^ [12,13,14,15]. These experiments indicate that G26C is more accessible and easily modified than G22C.

### 3.2. Single Channel Analysis of the Effects MTS Modifications in G26C MscL Activity

**Figure 3:**
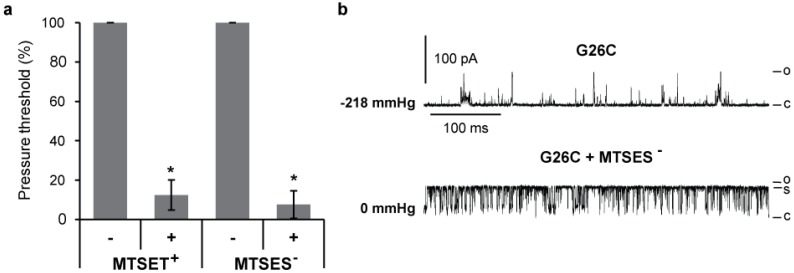
Effects of charged MTS modifications on G26C MscL gating. (**a**) The graph shows the changes in the pressure required to gate G26C MscL before and after treatment with charged MTS reagents MTSET^+^ and MTSES^−^ (n ≥ 3). Values were normalized to the pressure required to gate the channel before treatment. (**b**) The current traces of a typical patch clamp experiment in native membranes from giant spheroplasts are shown before (**top**) and after (**bottom**) the addition of MTSES^−^ to the patch chamber buffer. The pressures at which the traces were recorded are indicated at the left in mmHg. The letters C, S and O on the right of the traces indicate closed, sub-conductive and open states respectively (* p ≤ 0.004 in Student t-test paired control *vs*. treated).

Patch clamp allows quantification of the effects of MTS modifications on single channel activities. Thus, we assayed G26C MscL activity by patch clamping native membranes of giant spheroplasts, as previously described [16]. MscL channels were activated by applying negative pressure to the patch; the minimal pressure required to gate the channel, defined as pressure threshold, was measured [26,28]. The changes in pressure threshold before and after treatment with the charged MTS reagents MTSES^-^ and MTSET^+^ are shown in Figure 3(a); the introduction of either positive or negative charges at the G26 position greatly reduces the pressure required to gate the channel. The mean pressure threshold of G26C MscL before treatment was −152.3 ±12.6 mmHg and after treatment 13.3 ± 6.3 (n = 8). A representative trace of G26C MscL in such an experiment is shown in Figure 3(b), where the current from single channel activity from the same patch is shown before (upper trace) and after (lower trace) treatment with MTSES^-^. The pressure at which channel activity was recorded is shown at the left of each trace in mmHg. Modifications by the negatively charged MTSES^-^ not only made the channel gate in a pressure-independent manner, but also the kinetics were affected since the channel open dwell time increases and a more stable sub-conductive state is observed. 

These data are consistent with previous traces obtained for the positively charged MTSET^+^ modification [13], but they contrast studies with G22C where complete openings were never observed for MTSET^+^ or MTSES^-^ treated G22C MscL [15], and the channels were flickery, only opening briefly to substates. Hence, collectively these data demonstrate that the introduction of a positively or negatively charged molecule at position 26, but not 22, stabilizes the channel in an open state. 

### 3.3. Hydrophobic MTS Modifications in G26C MscL, Closing MscL Nanovalve

Another desirable feature for a gated nanovalve is reversibility since it opens the possibility to use the channel as a nano-switch. Under the hypothesis that hydrophobic substitutions in G26C MscL would lead to channels with an increased gating threshold, in opposition to the effects of charged substitutions, we also studied the effects of three MTS hydrophobic reagents in G26C MscL activity by patch clamp. 

**Figure 4:**
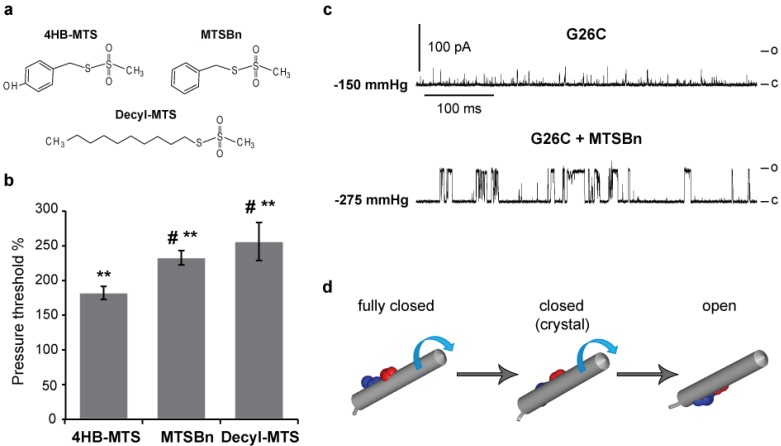
Effects of hydrophobic MTS modifications in G26C MscL gating. (**a**) Structures of the hydrophobic MTS reagents 4-HB-MTS, MTSBn and Decyl-MTS are shown. (**b**) A bar graph showing the pressure threshold changes of G26C MscL when treated with 50 µM 4-HB-MTS, 100 µM MTSBn or 10 µM Decyl-MTS. The changes in the pressure required to gate the channels before and after treatment with hydrophobic MTS reagents are expressed as a percentage of the pressure threshold of the same patch before treatment (100% means no change) (n ≥ 13). (**c**) Typical single channel activities of G26C MscL in native membranes from giant spheroplasts are shown to illustrate the changes in kinetics and pressure threshold after a hydrophobic substitution with MTSBn. The numbers on the left of each trace is the pressure at which the patch was held at the time of the recording. (**d**) A graphic depicting TM1 and the modeled positions of residues G22 and G26. TM1 is represented in a fully closed, closed (crystal structure) and open states. The proposed clockwise rotation of TM1 during MscL gating is represented by the blue arrows. ** p ≤ 1.8 × 10^−7^ in Student t-test paired control *vs*. treated, # p ≤ 0.03 in Student t-test unpaired *vs*. 4HB-MTS.

The structures of the tyrosine-like 4HB-MTS, the phenylalanine-like MTSBn and the long fatty acid-like Decyl-MTS are shown in Figure 4(a). Again the pressures needed to gate the channel before and after treatment were measured. As anticipated, all three hydrophobic substitutions caused an increase in the pressure threshold from 75 to 150% as shown in Figure 4(b), with average pressure before treatment of −88.5 ± 5.5 (n = 49). Interestingly, more hydrophobic substitutions led to greater increases in the pressure threshold needed to gate the channel. Another obvious effect of hydrophobic substitutions can be observed in the trace shown in Figure 4(c), where current traces from G26C MscL before and after MTSBn treatment are shown. There is again a drastic effect in channel kinetics with more full openings and longer open dwell times achieved after treatment with MTSBn (lower trace). Note that the pressure threshold after MTSBn modification significantly increased in this trace. A current model predicts a clockwise rotation of TM1 while the channel gates; the model also predicts that residue G26 would be at the constriction point when the channel is fully closed, yet facing TM2 and the membrane upon opening [2,29,30]. The previous observation that substitutions at G26 with charged MTS reagents stabilize channels in an open sub-state, not a fully opened state [13], and the current finding that the hydrophobic substitutions favor a fully open state of the channel are consistent with this model (see Figure 4(d)). 

### 3.4. Calcein Release through MscL Nanovalves from Liposomes: A Model for Drug-Delivery

Finally, we tested the relative efficiency of these two MscL mutants to act as gated nanovalves in a liposomal flux system. We measured the flux of the fluorescent dye calcein through MscL as an indication of MscL activation. Two different modes of activation were used to gate MscL: treatment with charged MTS reagents and UV light pulses. For the efflux experiments we used a truncated version of G26C MscL, G26C (Δ110-136) MscL, because it improves protein yield during purification (see Table 1). Although the protein yield is lower for G26C (Δ110-136) than G22C MscL, enough protein can be purified for reconstitution experiments. Briefly, purified G26C (Δ110-136) or G22C MscL protein was incorporated in synthetic lipid vesicles loaded with the self-quenching fluorescent dye calcein as described in Materials and Methods. 

**Figure 5:**
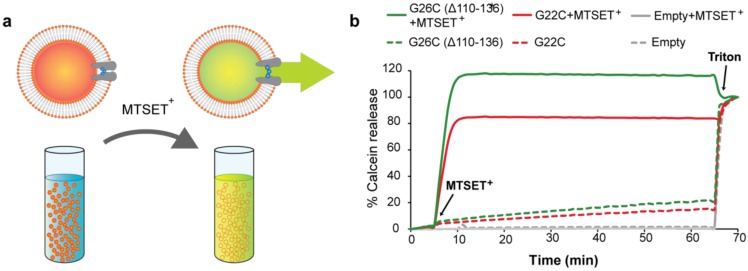
Efflux efficiency through G22C and G26C (Δ110-136) MscL as activated nanovalves in artificial liposomes. (**a**) The scheme represents the efflux of the self-quenching dye calcein from MscL proteo-liposomes gated by MTSET^+^. Calcein released through the channel is diluted giving a fluorescence signal. (**b**) Comparison of the efficiency of calcein efflux through G22C and G26C (Δ110-136) MscL gated by modification with the charged MTS reagent MTSET^+^. Proteo-liposomes filled with calcein and containing no protein (empty), G22C or G26C (Δ110-136) MscL were treated with 1 mM MTSET^+^. Fluorescence of the calcein released through MscL nanovalve was plotted against time. Triton was added at the end of the experiments to control for the liposomes loading with the dye.

Because of its self-quenching property, calcein does not fluoresce inside the lipid vesicles where its concentration is high, but its fluorescence can be measured when it is released and diluted in the external solution Figure 5(a). Calcein efflux could be elicited with treatment with MTSET^+^ from liposomes containing G22C and G26C (Δ110-136) MscL protein as shown in Figure 5(b). Consistent with the *in vivo* results, calcein efflux through G26C MscL was more efficient than G22C MscL at the same protein:lipid ratio. Both charged MTS reagents showed a similar trend with higher total fluorescence released from G26C (Δ110-136) than G22C MscL liposomes; G26C (Δ110-136) flux was 26.12 ± 5.5 % (n = 4) (p ≤ 0.002, Student t-test unpaired) higher than G22C for MTSET^+^ and 25.6 ± 6% (n = 3) higher for MTSES^−^ (p ≤ 0.007, Student t-test unpaired). No calcein release was observed after MTSET^+^ was added to vesicles containing no protein. All liposomes were loaded with the dye as confirmed by the fluorescence observed after lysis with detergent. Note that for experiments in which 100% of calcein is released a slight drop in the fluorescence is observed upon addition of triton probably due to the dilution of the dye.

**Figure 6:**
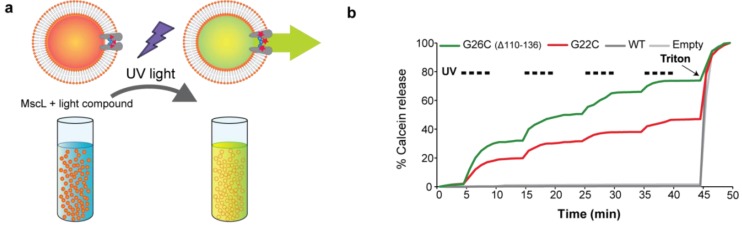
Efflux efficiency through G22C and G26C (Δ110-136) (MscL light activated nanovalves in artificial liposomes. (**a**) The scheme represents the efflux of the self-quenching dye calcein from MscL proteo-liposomes. MscL channels modified with a light-compound are gated by pulses of UV light. Calcein released through the channel is diluted giving a fluorescence signal. (**b**) Calcein efflux through G26C (Δ110-136) and G22C MscL modified with a light sensitive compound was measured. Proteo-liposomes filled with calcein containing no protein (empty), WT, or light-compound modified G22C or G26C (Δ110-136) MscL were treated with UV light (336 nm) pulses of 5 min of duration. Triton was added at the end of the experiments to control for the liposomes loading with the dye.

A second gating modality for MscL pore mutants, UV light, was used in this flux assay. In this case the purified protein from MscL mutants was modified with the light-sensitive compound described in Materials and Methods before being incorporated into liposomes. As shown in Figure 6(a) after UV treatment, the light sensitive compound becomes charged (zwitterionic) eliciting the gating of the modified MscL mutants [20,21]. Both G22C and G26C (Δ110-136) MscL showed activation by UV light pulses as indicated by the increase in fluorescence after the stimuli (Figure 6(b)). The activation by light was consistently more efficient for from liposomes containing G26C (Δ110-136) than G22C MscL, with the later achieving only 55.2 ± 4.6% (n = 3) (p ≤ 0.0007 Student t-test unpaired) of G26C total flux; no efflux was observed from liposomes containing WT MscL. These results suggest that MscL activation is more efficient when using G26C (Δ110-136) mutant than G22C for both modes of activation. 

### 3.5. The Many Dimensions for Designing Device-Specific MscL-Based Triggered Nanovalves

Many characteristics of *E. coli* MscL make it an ideal candidate for its use as a nanovalve for targeted drug-release. It has a small protein size (136aa) [31], is amenable for solubilization and reconstitution [28], and its large pore size allows the passage of large molecules upon gating [4]. It can be purified in large quantities *in vivo* or in cell-free system [17], or even chemically synthesized [18], yet once in membranes it assembles into a functional complex. In addition, the nanovalve is extremely malleable, allowing one to design it for specific purposes. The channel can be gated by introduction of a single charge in the pore region in what is known as the hydrophobic lock; this allows one to engineer it to respond to different modalities, including light and pH, through modifications with light or pH sensitive compounds that become charged upon stimulation [19,20,21]. The permeation of specific charged chemicals can be enhanced [22], and the pore size can even be reversibly adjusted [23] with further engineering. In addition, G26C MscL has recently been shown to express and function in mammalian cells, thus serving as controlled delivery route of substances into the cytoplasm [32]. Although most of the above mentioned experiments have been performed using G22C MscL [19,20,21], here we propose the use of another site in the channel hydrophobic lock, G26. We find that G26C MscL nanovalve is more amenable to modifications and consistently shows greater efficiency that G22C *in vivo*, in patch clamp, and in efflux experiments. One of the interesting differences between the two mutants, evidenced by patch clamp, is the kinetics of the modified channels. While the G26C MscL achieves a stable open sub-state when modified with a charged MTS reagent (Figure 3(b)), G22C has been shown to only achieve very short and unstable openings [15]. Depending on the device and specific use of the MscL nanovalve, these differences in opening size and stability could be crucial, as for example in the delivery of larger molecules for a drug delivery system. 

Here, for the efflux experiments, we use a truncated version of G26C MscL, G26C (Δ110-136) MscL, because this truncation increases the protein yield (Table 1). It has been described in the early characterization of MscL, that this deletion has no mayor effect in MscL channel activity [26]. In a recent publication of Li *et al*. truncated versions of the MscL protein were used in efflux experiments and showed a behavior consistent with the full length constructs [29]. In addition comparisons between a homolog MscL from *S. Aureus* studied with or without the same c-terminal truncation showed that channel activities were similar [33,34]. 

Another interesting finding is that the pressure required to gate G26C MscL showed an increase of 80 to 150% when substituted with hydrophobic MTS reagents. Again, a much smaller change in pressure threshold, of only about 25%, was reported for hydrophobic substitutions of G22C MscL [15]. The ability to inhibit G26C gating by the introduction of such a hydrophobic substitution could be an advantage in the design of a nano-switch, e.g., the modification of a hydrophobic compound that becomes zwitterionic or charged upon changes in the pH or light may be more tightly regulated if placed at the G26 site. 

**Figure 7:**
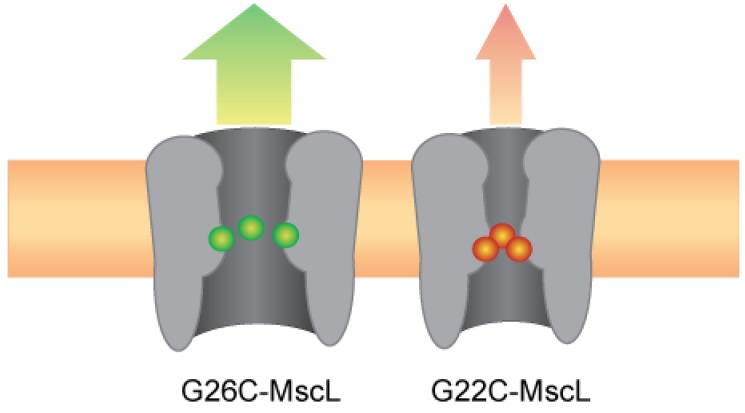
Improved efficiency of the MscL-based nanovalve; G26 is more accessible and stabilizes an open state.

## 4. Conclusions

The significance of this study is that we compared the efficiency of G26C-MscL as a gated nanovalve in side by side experiments with G22C-MscL and found that G26 appears to be a preferable site for modification than G22 for designing a MscL-based triggered nanovalve. We demonstrate this in a liposome efflux system by using two different gating mechanisms: charged MTS substitutions and a UV light (Figure 5, Figure 6). Our results suggest two likely reasons for the higher efficiency of G26C-MscL nanovalve: the more stable and larger channel openings achieved upon modification and a higher accessibility to the site by the modifying compounds and stimuli. 
